# Primordial soup or vinaigrette: did the RNA world evolve at acidic pH?

**DOI:** 10.1186/1745-6150-7-4

**Published:** 2012-01-20

**Authors:** Harold S Bernhardt, Warren P Tate

**Affiliations:** 1 Department of Biochemistry, University of Otago, P.O. Box 56, Dunedin, New Zealand

**Keywords:** RNA world, evolution, acidic pH, protonated base pairs, RNA triple helix, tRNA

## Abstract

**Background:**

The RNA world concept has wide, though certainly not unanimous, support within the origin-of-life scientific community. One view is that life may have emerged as early as the Hadean Eon 4.3-3.8 billion years ago with an atmosphere of high CO_2 _producing an acidic ocean of the order of pH 3.5-6. Compatible with this scenario is the intriguing proposal that life arose within alkaline (pH 9-11) deep-sea hydrothermal vents like those of the 'Lost City', with the interface with the acidic ocean creating a proton gradient sufficient to drive the first metabolism. However, RNA is most stable at pH 4-5 and is unstable at alkaline pH, raising the possibility that RNA may have first arisen in the acidic ocean itself (possibly near an acidic hydrothermal vent), acidic volcanic lake or comet pond. As the Hadean Eon progressed, the ocean pH is inferred to have gradually risen to near neutral as atmospheric CO_2 _levels decreased.

**Presentation of the hypothesis:**

We propose that RNA is well suited for a world evolving at acidic pH. This is supported by the enhanced stability at acidic pH of not only the RNA phosphodiester bond but also of the aminoacyl-(t)RNA and peptide bonds. Examples of *in vitro*-selected ribozymes with activities at acid pH have recently been documented. The subsequent transition to a DNA genome could have been partly driven by the gradual rise in ocean pH, since DNA has greater stability than RNA at alkaline pH, but not at acidic pH.

**Testing the hypothesis:**

We have proposed mechanisms for two key RNA world activities that are compatible with an acidic milieu: *(i) *non-enzymatic RNA replication of a hemi-protonated cytosine-rich oligonucleotide, and *(ii) *specific aminoacylation of tRNA/hairpins through triple helix interactions between the helical aminoacyl stem and a single-stranded aminoacylating ribozyme.

**Implications of the hypothesis:**

Our hypothesis casts doubt on the hypothesis that RNA evolved in the vicinity of alkaline hydrothermal vents. The ability of RNA to form protonated base pairs and triples at acidic pH suggests that standard base pairing may not have been a dominant requirement of the early RNA world.

**Reviewers**

This article was reviewed by Eugene Koonin, Anthony Poole and Charles Carter (nominated by David Ardell).

## Background

The concept of an RNA world - an early stage of evolution where RNA functioned as both gene and catalyst - has wide, though certainly not unanimous, support among those who study the origin of life. However, any such evolutionary model needs to be firmly rooted in an understanding of likely primordial physical and chemical Earth conditions [1]. The hypothesis that life may have emerged during the Hadean Eon 4.3-3.8 billion years ago [2] is supported by the discovery of zircon crystals that suggest the presence of liquid water and continental crust on the Earth as early as 4.4 billion years ago, or within 150 million years of the Earth's formation [3]. A projected high level of atmospheric CO_2 _is proposed to have produced an acidic ocean of the order of pH 3.5-6 [4,5] (for the purpose of our hypothesis we have chosen pH 4-5 as acceptable mid-range values). The pH is inferred to have subsequently risen to ~6.8 as CO_2 _levels gradually decreased during the Hadean Eon; in parallel, the ocean temperature may have dropped from ~100 to ~70°C [5]. Alternatively, air and ocean temperatures may have become clement soon after the Earth's formation [6,7].

**Figure 1:**
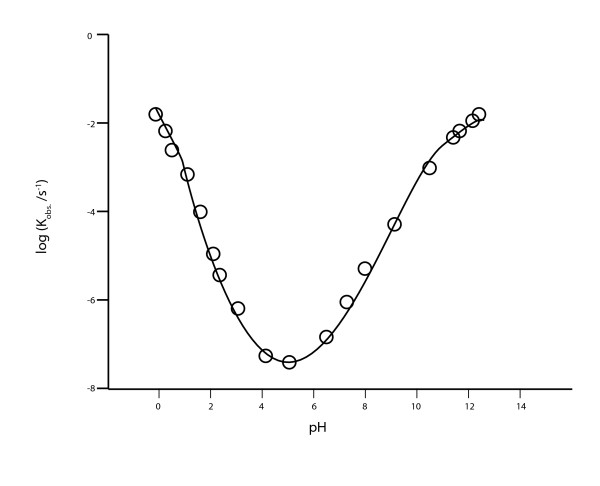
**The RNA phosphodiester bond is most stable at pH 4**-**5 at 90°C**. Hydrolysis of the dinucleoside 3',5'-UpU at 90°C as a function of pH. Figure reprinted with permission from [15] Oivanen *et al. *^©^1998 American Chemical Society; data used with permission from [10] Järvinen *et al. *^©^1991 American Chemical Society.

Michael Russell and colleagues have proposed that life arose within alkaline (pH 9-11) deep-sea hydrothermal vents similar to those of the Lost City [8,9], with the interface with the acidic ocean forming iron sulfide structures that functioned as proto-membranes, across which a proton gradient occurred sufficient to drive the first metabolism (see also [6]). However, one difficulty with this attractive scenario is the instability of the phosphodiester bond of RNA at alkaline pH [10] (Figure 1), suggesting that RNA is more likely to have evolved in an environment of lower pH. Indeed a number of investigators in simulated experiments have isolated *in vitro*-selected ribozymes with activity at acid pH (sometimes optimal), and have suggested as a result that the RNA world may have evolved in such an acidic milieu, for example Jayasena and Gold [11]:

"An infinite number of conditions may be chosen to explore the versatility of RNA for catalysis, and to that list one must now add low pH... The prebiotic "RNA world" may have been more robust than is suggested by the limited reactions catalyzed thus far by RNAs under rather more standard laboratory conditions" [11].

Similarly, Burke and Hoffman [12] point out that their discovery of an RNA that binds coenzyme A and other adenosine analogues with a pH optimum of 5, suggests that such harsh conditions may be compatible with those existing in an RNA world. Echoing Jayasena and Gold, they argue that this makes the RNA world theory more robust than if the supporting evidence came only from mild laboratory conditions. Miyamoto and colleagues [13] note that acidic seawater or acidic clay layers are included in discussions of possible environments on the prebiotic Earth, and that depurination of deoxyribonucleosides occurs at acid pH, making DNA more labile than RNA under these conditions. They suggest that a first step in developing a self-replicating RNA might have been a ligase ribozyme functional in acidic conditions, similar to the one they isolated by *in vitro *selection [13,14].

**Figure 2:**
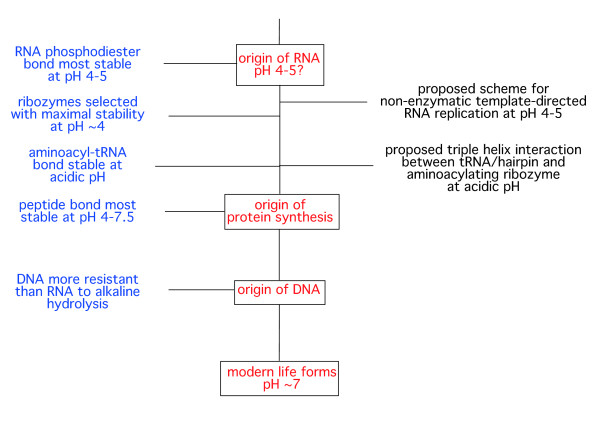
**Proposed timeline of early evolution from the origin of RNA (pH 4**-**5) to modern life forms (pH ~7)**. On the right of diagram are two proposed RNA world mechanisms compatible with acidic pH (see section 'Testing the hypothesis' for details).

The following observations are consistent with the hypothesis that the RNA world was compatible with an acidic pH environment. The supporting observations are presented according to their proposed occurrence chronologically, in parallel with a proposed gradual increase in pH (Figure 2).

### The phosphodiester bond of RNA is most stable at pH 4-5 at 90°C

RNA is susceptible to alkaline hydrolysis at pH > 6, whereas, in contrast, acid hydrolysis only occurs at pH < 2 [15]. As shown in Figure 1, Järvinen and colleagues [10] found that the rate of phosphodiester bond hydrolysis within the RNA dinucleoside 3',5'-UpU is lowest at pH 4-5 at 90°C, especially relevant to models that propose a hot Hadean ocean [5]. Prior to the emergence of coded protein synthesis - and the evolution of proteins able to bind to and protect RNA - an acidic environment may have conferred stability to RNA necessary for its evolution.

### Ribozymes with maximal activity at acidic pH have been isolated by *in vitro *selection

**Figure 3:**
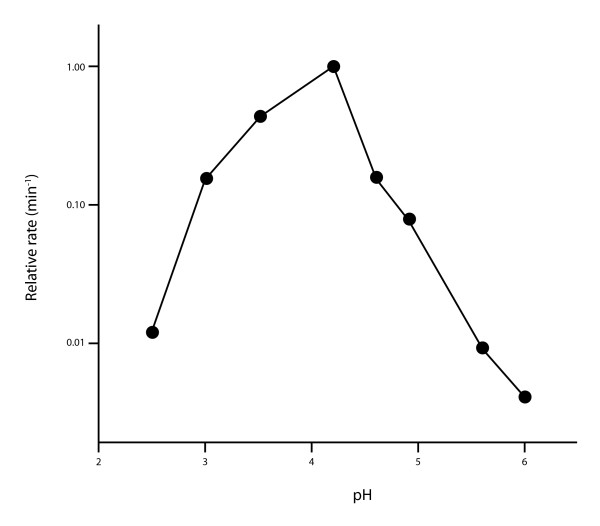
**A self-cleaving ribozyme isolated by *in vitro*-selection has maximal activity at pH 4.2**. The rate of cleavage obtained at each pH value was normalized to the maximum rate measured at pH 4.2. Figure adapted with permission from [11] Jayasena and Gold 1997 ^©^1997 National Academy of Sciences, U.S.A.

A number of ribozymes with a range of catalytic activities have been isolated by *in vitro *selection at acidic pH. In some ways this is not surprising, as the RNA bases A, C and G have *pK*_a_s between 2 and 4, and so catalytic activity utilising this ionization could be expected at low pH. Ribozymes active at acidic pH - sometimes maximally - catalyse RNA ligation [13,14,16], self-cleavage [11] (Figure 3) and amino acid-activation (similar to the synthesis of aminoacyl-adenylates by modern protein aminoacyl-tRNA synthetases) [17]. This is a limited number of activities but all would have been important in the context of an RNA world. As discussed, Miyamoto and colleagues have argued that RNA ligation may have been a forerunner to self-replication [13].

### The aminoacyl-tRNA bond is more stable at acidic pH

**Figure 4:**
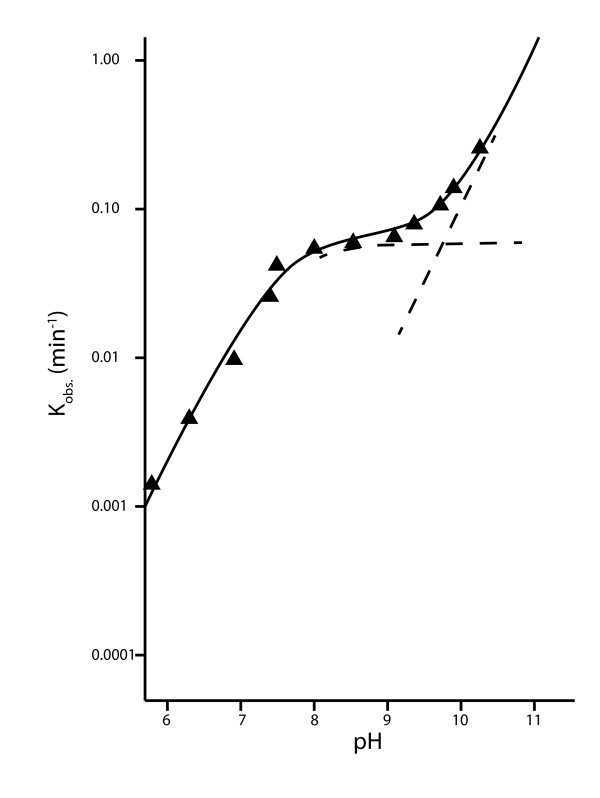
**Rate of hydrolysis of aminoacyl**-**tRNA decreases with pH over the range pH 6**-**11**. Rate of hydrolysis of leucyl-tRNA at 37°C and ionic strength 0.30 (the break in the curve between pH 8-10 represents the shift from rate-determining hydroxide attack on the ammonium acid ester cation to rate-determining attack on the free amino acid ester). Figure adapted with permission from [22] Wolfenden ^©^1963 American Chemical Society.

The ease with which ribozymes able to catalyze self-aminoacylation or aminoacylation *in trans *have been isolated by *in vitro *selection suggests this is a reaction at which RNA is particularly adept [18], and therefore promiscuous aminoacylation was likely a hallmark of the RNA world. If so, this was fortunate, as aminoacylation of tRNA - and the hairpin precursor from which it is thought to have evolved [19,20] - was probably essential for the evolution of coded ribosomal protein synthesis [21]. However, the mixed phosphate anhydride bond of aminoacyl-tRNA is particularly unstable at neutral and alkaline pH [22,23]. This is not the case at acidic pH; as illustrated in Figure 4 by the hydrolysis of leucyl-tRNA as a function of pH, the rate of hydrolysis decreases with decreasing pH from pH 11 to 6 - the lowest value measured [22]. Because of the greater stability of this bond at acidic pH, typically aminoacylated tRNAs are purified at pH 4.5 (U. Varshney, pers. commun.) and analyzed by PAGE at pH 5 [24]. Stabilization of the aminoacyl-RNA bond would have been critical to the early evolution of coded protein synthesis, as unlike in the modern system, there would have been no protection of this bond by the protein elongation factor EF-Tu, which transports aminoacyl-tRNA to the ribosome [25].

### The peptide bond is most stable over the range pH 4-7.5

Pascal and colleagues [26] have calculated the theoretical influence of pH on the ratio of equilibrium constants for peptide bond formation. Perhaps surprisingly, the results indicate that the peptide bond is stable over the range pH 4-7.5, with optimum stability at pH 6. This is consistent with our current understanding that protein synthesis arose within the RNA world, which, according to our hypothesis, would have been when conditions were still relatively acidic (see Figure 2).

### DNA is more resistant than RNA to alkaline hydrolysis

While RNA is prone to alkaline hydrolysis at pH > 6 [15], the 2'-deoxy sugar of the DNA backbone is much more resistant [27]. The presence of the vicinal 2',3'-hydroxyl groups on the ribose of RNA means that alkaline hydrolysis proceeds via the formation of an intermediate cyclic 2',3'-phosphonucleoside, whereas deoxyribose lacks the 2'-OH group required to form this intermediate. It is possible that the rise in ocean pH as the Hadean Eon progressed was partly responsible for driving the transition from an RNA to a DNA genome. The greater stability of the peptide bond [26] compared with DNA [13] at acidic pH supports the conjecture that proteins evolved before DNA [28].

## Presentation of the hypothesis

We propose that the RNA world evolved in an acidic milieu. Stability was critical for evolution: the central tenet of our hypothesis is that chemical identities, including molecular species and bonding interactions, require stability in order for evolution to take place. The fundamental importance of stability can be seen in the core structure of RNA, where it is possible that base pair interactions afforded nucleotide bases within the interior of double-stranded helical regions protection against chemical attack; RNA hairpins are remarkably stable in comparison with single-stranded RNA

[29]. An acidic environment would have further increased the stability of RNA and therefore the chances for its evolution. Likewise, acidic pH would have enhanced the half-life of the aminoacyl-(t)RNA bond, increasing the chances for the evolution of protein synthesis.

## Discussion

These ideas were first presented by HSB at ISSOL 2011 in Montpellier France. At this conference, Jeremy Kua (Kua and Bada, 2011; [30]) presented complementary work on the stability of RNA drawn from a comparative analysis of published data of the stability of *(i) *the ribose sugar, *(ii) *cytosine pyrimidine base, and *(iii) *the phosphodiester bond, for various environments proposed for the origin of life (pH 3.5-10). This included both acidic and alkaline hydrothermal vents and an acidic ocean. They concluded from this analysis that RNA has maximum stability at 0°C at pH 5.5 and at 75°C at pH 5 [30], consistent with our findings.

Further support for the hypothesis comes from recent work carried out by Perez-Jimenez and colleagues [31], who used phylogenetic analysis to infer the sequences of thioredoxin protein enzymes from extinct organisms. When produced in the laboratory, the 'oldest' of the reconstructed enzymes - proposed to date back 4 billion years - have considerably higher activity at pH 5 than their modern counterparts. Although their results throw light on the environment during the early evolution of proteins (supporting our hypothesis that this occurred during the Hadean Eon) as opposed to the RNA world, their findings are consistent with our proposal that conditions at this later time were still relatively acidic (see Figure 2). In contemporary systems, thioredoxin is both an intracellular and secreted protein [32]. If this were also the situation during the early evolution of thioredoxin, it suggests that both the external and intracellular environment were more acidic at this time 4 billion years ago than they are today [31]. This is possible if at the time thioredoxin evolved the cell membrane was much 'leakier' than today, and was therefore an imperfect barrier to protons. A useful comparison can be made with the acidophilic eubacterium *Acetobacter aceti (A. aceti) *that converts ethanol in wine into acetic acid in the industrial production of vinegar and can function with an intracellular pH as low as 3.9 [33]. Because of the high membrane-permeability of acetic acid, *A. aceti *has limited ability to maintain its internal pH and so at times experiences the most acidic cytoplasmic pH of any known organism: when grown on ethanol, the external pH and internal pH decrease in parallel from 6.2 to 3.5 and from 5.8 to 3.9, respectively [33]. In view of the probable close relationship between external and intracellular pH in the early evolution of life, the greater stability at acid pH of RNA and key RNA bonds utilized in modern biology supports our hypothesis that (membrane-encapsulated) RNA world evolution took place in an acidic environment.

## Testing the hypothesis

The following two proposed RNA world activities are compatible with an acidic milieu, and are open to experimental testing. While not proving the hypothesis, demonstration of the feasibility of either or both of these mechanisms (and the acidic conditions required) would give added weight to our proposal of an RNA world evolving at acidic pH (see Figure 2 for a possible chronology of the proposed mechanisms).

### A possible scheme for non-enzymatic template-directed RNA replication at pH 4-5

**Figure 5:**
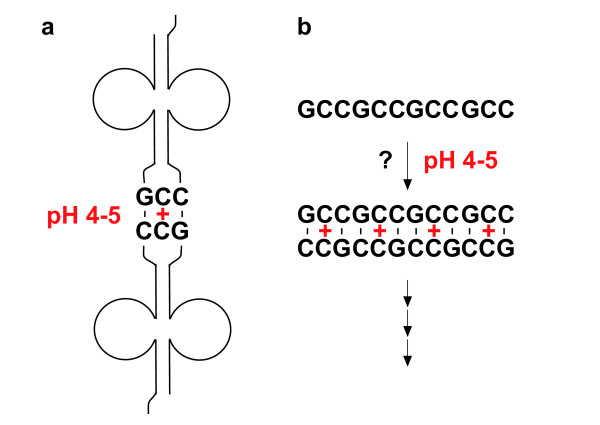
**A possible scheme for non-enzymatic RNA replication at pH 4**-**5**. **(a) **tRNA^Gly(GCC) ^forms dimers through its GCC anticodon due to hemi-protonation of the central cytosine at pH 4-5 [37]**(b) **At pH 4-5, C-rich (GCC)*_n _*sequences might produce C-rich duplicates by non-enzymatic replication. Base pairs involving hemi-protonated cytosines are indicated by (+).

Prior to the emergence of an RNA replicase, non-enzymatic replication of shorter sequences of RNA may have occurred. It has proven difficult to find a pair of complementary oligonucleotides each of which will act as an efficient template for the other, allowing repeated amplification of the two sequences [34]. Work in this area has focused on C-rich sequences such as the RNA oligonucleotide CCGCC [35] and DNA oligonucleotide (CCCG)_3_CC [36], which make particularly good templates (this work has used the activated RNA nucleotide analogues 5'-phosphoro (2-methyl)-imidazolides, which - unlike standard RNA nucleotides - undergo efficient and regiospecific template-directed incorporation into a copy strand). At neutral pH however, C-rich sequences produce G-rich copies that tend to form higher-order structures that inhibit further replication [34]. Non-standard base pair interactions that can occur at acidic pH suggest a possible solution to this dilemma. As shown in Figure 5a, *Escherichia coli (E. coli) *tRNA^Gly(GCC) ^forms dimers through its quasi-self complementary GCC anticodon, but only at pH 4-5 [37]. Romby and colleagues proposed that, at this acidic pH, the GCC anticodon is able to self-pair through the sharing of a proton between the two central cytosines, forming a C-C(+) hemi-protonated base pair. It would therefore appear possible that, as illustrated in Figure 5b, hemi-protonated linear GCC repeat sequences could serve as templates to produce identical copies at pH 4-5. If so, this might have provided a mechanism for successful non-enzymatic replication at the dawn of the RNA world, as both template and copy strands are similarly (equally) C-rich. DNA sequences consisting of GCC repeats form hairpins even at neutral pH [38], and these should be stabilized in acidic conditions by protonated C-C(+) base pairs (RNA hairpins probably played an important role in the early evolution of the RNA world due to their resistance to thermal and chemical degradation). Similarly, formation of protonated C-A(+) base pairs at acidic pH [39-43] suggests that RNA sequences consisting of GCC repeats might also template GAC repeat sequences or mixed GCC/GAC sequences under these conditions.

### A mechanism for specific aminoacylation of RNA hairpins and ancestral tRNA

**Figure 6:**
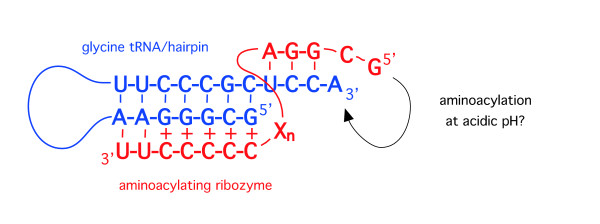
**A possible mechanism for aminoacylation specificity in the RNA world**. Specificity could have been through a triple helix interaction at acidic pH between the aminoacyl stem of a tRNA/precursor hairpin (shown here as the glycine tRNA sequence) *(blue) *and an aminoacylating ribozyme comprised of an active variant of the 5-nucleotide ribozyme from [48] fused to a 3' polypyrimidine 'tail' *(red)*. See text for further comments and references. X_n _= nucleotide linker sequence. Base triples involving protonated cytosines are indicated by (+).

With an RNA world at acidic pH, a possible mechanism for the specific aminoacylation of tRNA (and its hairpin precursor [19,20]) can be suggested. Modern protein aminoacyl-tRNA synthetases achieve specificity through recognition of particular sequences of nucleotides in the tRNA aminoacyl stem adjacent to the 3' CCA terminus where the amino acid is attached, which has been termed the RNA operational code [44]. Di Giulio has proposed that tRNA arose by the duplication and ligation of a hairpin approximately half the length of the contemporary tRNA molecule [19]. Due to the symmetry of base pair interactions, the RNA operational code in tRNA would also have been present in the precursor hairpin, and conserved in the transition from hairpin to tRNA [20]. Prior to the evolution of coded protein synthesis in the RNA world, aminoacylation would have been catalyzed by ribozymes. Recognition of the RNA operational code nucleotides by modern protein synthetases is through the interaction of these nucleotides with specific amino acid residues. Recognition by a ribozymal synthetase could have instead been through either *(i)* tertiary interactions, *(ii) *base pairing between ribozymal nucleotides and nucleotides of the operational RNA code following strand-separation of the tRNA/hairpin aminoacyl stem, or *(iii) *formation of a base pair-specific triple-helix interaction with the tRNA/hairpin aminoacyl stem, which includes the RNA operational code nucleotides. Such a pyrimidine triple helix - *pyrimidine-purine*-pyrimidine, with the Watson-Crick helix in italics - only forms at acidic pH, with the third polypyrimidine strand forming parallel Hoogsteen interactions with the polypurine strand within the major groove of the Watson-Crick double helix ([45]; see also [46]). Thermal dissociation studies have shown that, in DNA triple helixes at least, a single purine-pyrimidine swap in the Watson-Crick double helix can be accommodated within the triple helix, albeit with a slight decrease in stability for the resulting triple helix [47]. The RNA sequence analysed in [45] forms a triple helix with 7 base triples at pH 4.3, whereas the DNA triple helices containing single mismatches form stable structures at pH 5.6 [47]. Michael Yarus and colleagues have produced a 24-nucleotide self-aminoacylating ribozyme possessing only three conserved nucleotides [18] and this was subsequently truncated to a mere 5-nucleotide ribozyme able to aminoacylate a 4-nucleotide RNA substrate *in trans *[48]. This reaction suggests a possible mechanism for the specific recognition of the aminoacyl stem of tRNA or its hairpin *without *the requirement for strand-separation: as illustrated in Figure 6, a polypyrimidine stretch of the aminoacylating ribozyme could specifically recognize the operational RNA code nucleotides in the tRNA/hairpin aminoacyl stem through base-pair specific interaction with the purine-rich 5' strand at pH 4-5. Supporting this possibility, the analogous (5') strand of the aminoacyl stem of *E. coli *tRNA^Gly(GCC) ^(we have previously proposed tRNA^Gly ^was the first tRNA to evolve [20]) contains the strikingly purine-rich sequence GCGGGAA [49]; a number of other tRNAs have a similarly purine-rich 5' strand [49]. Different purine-rich sequences embedded in the aminoacyl stems of hairpins/tRNAs could have been recognized by complementary polypyrimidine sequence 'tails' of ribozymes able to catalyse the attachment of different amino acids. These purine-rich sequences may have been the forerunners of the operational RNA code, recognized today by protein aminoacyl-tRNA synthetases.

## Implications of the hypothesis

### RNA world incompatible with alkaline deep-sea hydrothermal vent origin

The major implication of our hypothesis is that it suggests the evolution of RNA is unlikely to have occurred in the vicinity of an alkaline deep-sea hydrothermal vent as has been proposed [4,6,8,9], but rather took place within the acidic Hadean ocean itself (possibly in the vicinity of an acidic deep-sea hydrothermal vent or 'black smoker'), in an acidic volcanic lake [7], or comet pond [50]. While it is certainly possible that molecular evolution prior to the emergence of RNA could have occurred in the vicinity of an alkaline vent, it seems that in order for RNA to evolve, the proto-cell would have had to escape this alkaline environment. The argument from parsimony, however, would suggest that *all *early evolution took place in acidic conditions, with the energy required by the first life forms potentially provided by a protonmotive gradient across the proto-cell membrane [51,52], as the acidic environment interfaced with a slightly more alkaline interior (in fact, de Duve has made the observation that "the widespread use of protonmotive force for energy transduction throughout the living world today is explained as a legacy of a highly acidic prebiotic environment and may be viewed as a clue to the existence of such an environment" [53]). If life arose at high temperature, only the most stable RNA structures - such as protonated GC-rich hairpins - would have been resistant to degradation and thus survived. It seems likely that life emerged as soon as it became physically possible for it to do so.

### Alternative base-pairing schemes: evidence from modern tRNA secondary structure

A second implication of our hypothesis concerns the ability of RNA to form non-standard base pairs at acidic pH (Engelhart and Hud have also suggested the possible importance in early evolution of alternative base pairings, such as purine-purine and pyrimidine-pyrimidine duplexes [54]). The ability of C-C [37,43] and C-A [39-43] to form protonated base pairs at acidic pH suggests that standard base pairing may not have been an essential or exclusive requirement of the early RNA world. Error-prone replication of RNA in modern systems is due to the absence of error-correction mechanisms [55], and this was doubtless also the situation prior to the evolution of complex proteins. Replication ambiguity due to non-standard base pairing may have provided an additional means of increasing RNA sequence variation in the early RNA world.

We wondered whether the acidic intracellular pH that has been reported for some acidophilic archaebacteria might permit an increase in the number of C-C and C-A 'mismatches' in their cellular RNAs due to the potential ability of such mismatches to form stable protonated base pairs at acid pH (it has been shown that C-A(+) pairs at pH ≤ 5 are of the same order of stability as G-C and A-U pairs at pH 7 [40]). For our analysis we chose the tRNA gene-set (tRNome) from fourteen archaebacterial species: *i) *three acidophilic species reported to have an internal pH ≤ 6: *Thermoplasma acidophilum *[56] with a reported internal pH of 6.2 [57], *Ferroplasma acidarmanus *[58,59] with a reported internal pH of 4.9 ± 0.5 [60], and *Picrophilus torridus*, the sister species of which *(Picrophilus oshimae) *has a reported internal pH of 4.6 [61]; *ii) *one acidophilic species, *Sulfolobus solfataricus *[62], the sister species of which *(Sulfolobus acidocaldarius) *has a reported internal pH of 6.5 [63]; and *iii) *ten non-acidophilic archaebacterial species for which the internal pH has not to our knowledge been reported: *Aeropyrum pernix, Archaeoglobus fulgidus, Halobacterium sp., Methanobacterium thermoautotrophicum, Methanococcus jannaschii, Methanopyrus kandleri, Methanosarcina barkeri, Pyrobaculum aerophilum, Pyrococcus abyssi *and *Sulfolobus tokodaii*. These sequences were taken from a database supplied by Marck and Grosjean ([64]; C. Marck, pers. commun.); and crosschecked against the sequences in the Lowe tRNA Genomic Database [59]. We manually inspected 20 base pairs in the four stems of the tRNA cloverleaf secondary structure that are almost exclusively standard G-C, A-U and G-U interactions. These 20 base pairs comprised the aminoacyl stem (7 base pairs), anticodon stem (5), T stem (5) and D stem (3). The fourth D stem base pair 13-22 was not used as it contains a high proportion of base pair mismatches in all known non-mitochondrial tRNAs. Our findings were as follows: *i) Thermoplasma acidophilum *(reported internal pH 6.2) has 2 C-A 'mismatches' (plus a T-T mismatch), *Picrophilus torridus *(internal pH ~4.6) has 5 C-A mismatches (plus a T-T and an A-A mismatch), and *Ferroplasma acidarmanus *(reported internal pH 4.9 ± 0.5), has 10 C-A mismatches (plus a T-T mismatch) (interestingly, none of these species' tRNAs contains a C-C mismatch in the 20 examined base pairs). Of the remaining 11 species analyzed from *(ii) *and *(iii), Methanosarcina barkeri *had 2 C-A mismatches and only 2 others had a single C-A or C-C mismatch. Comparing the numbers of C-A and C-C mismatches in the tRNomes of the 3 species with an internal pH ≤ 6 with those in the remaining 11 species (with a reported or presumed internal pH ≥ 6.5), gave a significance of p-value = 0.003 using the one-sided Wilcoxon Rank Sum (Mann-Whitney U) test [65], indicating a relationship between a reported acidic internal pH and the presence of C-A and C-C 'mismatches' in tRNA secondary structure (*F. acidarmanus*, which at ten has the highest number of such 'mismatches' of the sample group, could have a more acidic intracellular pH than has been reported [66]). One would predict that the C-A 'mismatches' in the tRNAs of *P. torridus *and *F. acidarmanus *are protonated, although definitive proof of this would require experimental confirmation. Nonetheless, our findings suggest that an acidic intracellular pH may have allowed for an increase in the number of protonatable C-A pairs in the tRNomes of acidophilic archaebacteria with an acidic internal pH. It would therefore appear feasible that such protonated base pairs might have played a structural role in an RNA world at acidic pH.

## Conclusions

We have presented evidence that an acidic chemical environment is most compatible with the evolution of an RNA world. Acidic pH stabilizes key intra- and intermolecular RNA bonds, including those important for the evolution of protein synthesis, and facilitates additional protonated base interactions so that the behaviour of RNA under these conditions would not be constrained by the standard rules of base pairing. The diversity of stabilizing bonds provides additional potential mechanisms for sustaining the RNA world and initiating an RNA-protein breakthrough. Such a hypothesis casts doubt on previous proposals of an origin of life in the vicinity of alkaline hydrothermal vents.

### Note added in proof

Since submitting the manuscript, David Deamer has alerted us to two papers by his group that demonstrate nonenzymatic RNA synthesis [67] and nonenzymatic templated DNA replication [68] from non-activated monophosphate nucleotides at pH ~2-3 in the presence of a lipid matrix undergoing repeated cycles of wetting/drying. However, the formation of C-A and/or C-C base pairs was not reported for nonenzymatic templated DNA replication at acidic pH [68].

## List of abbreviations used

5' UTR: 5' untranslated region; EF-Tu: elongation factor thermo unstable; NMR: nuclear magnetic resonance; PAGE: polyacrylamide gel electrophoresis; *pK*_a _: acid dissociation constant; tRNome: complete set of tRNA genes in a genome.

## Competing interests

The authors declare that they have no competing interests.

## Authors' contributions

HSB formulated and developed the hypothesis. WPT provided original ideas and played a mentoring role. Both authors discussed ideas and wrote the manuscript.

## Reviewers' comments

### Referee 1: Eugene Koonin

Bernhardt and Tate discuss at length the possibility that the RNA World evolved at mildly acidic pH in the vicinity of acidic hydrothermal vents or volcanic lakes, as opposed to alkaline vents under the Martin-Russell scenario. I believe that they have a good case given that higher stability of RNA under acidic conditions is textbook knowledge whereas recent experiments added multiple observations of acidic optima for ribozyme-catalyzed reactions. To me, this article reads more like a review than a typical hypothesis because the rather obvious idea that acidic conditions could be more conducive for the evolution of an RNA world than alkaline conditions has been aired repeatedly over years (eg, their Ref. 11). However, this does appear to be the first detailed article that is fully dedicated to the acidic RNA World scenario, and in that capacity the article should become a useful and welcome addition to the origin of life literature.

### Referee 2: Anthony Poole

In this article, Bernhardt and Tate consider an important matter, often ignored in discussions of the plausibility of the RNA world hypothesis - the chemical environment most favourable to such a scenario. Based on an informative review of available biochemical literature, they argue that the RNA world, if it existed, would have been most favourable at acidic pH. They also note that peptide bonds -- but not DNA -- are optimally stable at acidic pH, suggesting that scenarios for early evolution where a physical environment is invoked should include consideration of this fact. The authors go on to argue that an acidic Hadean ocean, but not the more alkaline environments of Lost City, would be favourable to the emergence of an RNA world.

I think the authors are onto something here, but it would have been good to have seen some broader discussion on the possible environment. I am not sure that synthesis (as opposed to degradation) in the ocean is expected - if the authors believe it is, this is an important point that needs to be integrated into discussion. In this respect, I would be interested to hear the authors' thoughts on the recent proposal for pumice as a possible site for the origin of life [69]. This seems broadly compatible with the authors' suggestion, and has the advantage of not requiring the origin of life in an ocean environment where degradation might prevail over syntheses.

#### Authors' response

As implied in the manuscript, the authors consider the first step in the emergence of life to have been compartmentalization within lipid vesicles. This is in contrast to hypotheses invoking 'naked genes on the beach' - as proposed in Lathe's fast tidal cycling or PCR hypothesis [70,71] - or those invoking inorganic compartments alone, such as iron sulfide bubbles [4,6,8,9], but does not preclude catalytic mineral nanoparticles being entrapped within vesicles. Brasier's pumice hypothesis [69] invokes lipid vesicles held within pumice nanopores and is compatible with our favoured 'oil slick hypothesis' as proposed by Nilson [72], with life evolving beneath a skin of organic matter, endogenous or delivered by extraterrestrial means. Assembly of the first lipid vesicles could have occurred at the 'oil'-ocean interface, with the proton gradient between an acidic ocean and a more neutral vesicle interior providing a source of energy. A surface layer would have afforded the first life forms protection from UV radiation from the sun, in the absence of atmospheric oxygen and the resultant ozone layer. Pumice could have provided an absorption matrix for lipids within the organic material on the ocean surface. Wetting/drying cycles invoked for beached pumice - with the addition of a lipid matrix - even have a slight overlap with Lathe's fast tidal cycling/PCR hypothesis [70,71]. Brasier and colleagues [69] write that their floating pumice rafts would allow for transport between these two wet and dry environments.

I do overall think the case made by the authors is an important one, and deserving of further consideration, but I do wonder about one or two of the arguments. For instance, the statement in reference to the Järvinen paper [10] on p6 is a little surprising - here it is pointed out that phosphodiester bond hydrolysis of dinucleosides is lowest at pH 4-5 at 90°C, within the temperature range of the Hadean ocean. What worries me about this is that such temperatures do not seem particularly compatible with functioning RNA. Here the relevant property is not the conditions under which phosphodiester bond hydrolysis occurs but the conditions under which tertiary structure (and by proxy, RNA function) is stable. Given that tertiary structure is not known to be stable at such high temperatures, even in thermostable ribozymes [73], I suspect a much lower temperature would have been necessary for an RNA world. This might not be incompatible with the general conclusions drawn by the authors regarding an acidic environment for an RNA world -- some more careful consideration on the relationship to temperature might be valuable here: specifically, how much does lowering pH improve tertiary structure stability of RNAs?

#### Authors' response

As indicated by the reviewer, lower temperature conditions are not incompatible with our proposal of an acidic environment of the RNA world, and indeed RNA is stable at acid pH over a wide range of temperatures, with modest changes in pH for optimum stability (as shown by Kua and Bada [30], ribose, cytosine and the phosphodiester bond are most stable at 0 degrees celsius at pH 5.5). It is clear that the two main forces responsible for RNA secondary and tertiary structure, hydrogen bonding and Van der Waals (hydrophobic) interactions, would be diminished at high temperatures. Electrostatic interactions due to the formation of protonated base pairs [74] could increase tertiary structure stability at elevated temperatures, partly through interactions between protonated base pairs and the negatively charged phosphate backbone. Such an effect would be sequence-dependent. This would be similar to the increase in (hyper)thermophilic protein stability afforded by an increase in the number of charged surface residues [75].

Another case where a clearer argument on temperature could be made is in reference to ancestral reconstruction of thioredoxin enzymes, EF-Tu, and the discussion of protein enzymes from *A. aceti *that are thermostable and resistant to acidic pH (p11). These comparisons are weak, partly because such ancestral reconstruction is likely to be difficult, but mostly because, signal or no signal, they focus on protein, whereas the period the authors are looking at surely predates such proteins. In this respect, the work of Gouy and colleagues [76,77] may be more relevant; those authors looked at GC content of rRNA as a means of estimating the temperatures in which the Last Universal Common Ancestor existed. This work suggests a moderate temperature, though again -- and this is relevant to both protein and RNA ancestral reconstruction -- it is not possible to go further back than this hypothetical point, so, technical issues aside, it is difficult to say if this work sheds direct light on optimal temperature in an RNA world. Indeed, this point was made by Arrhenius and colleagues [78] in a letter that sought to correct a journalistic misinterpretation of Galtier and colleagues' work.

#### Authors' response

The authors completely agree with the thoughtful comments of the reviewer here, and this section has been removed from the final version of the manuscript.

I was intrigued by the discussion on p17 regarding the potential impact of an acidic cellular environment on protonated base pairs in tRNA from *A. aceti*, which is certainly an interesting line of enquiry. However, I was a bit surprised at how the paper ends - it seems rather abrupt, and lacks some concluding remarks or synthesis. I think adding a final conclusions-type section would greatly enhance readability.

#### Authors' response

An analysis of protonated base pairs in the bacterium *A. aceti *in an earlier version of the manuscript has now been replaced by an analysis of their occurrence in 14 archaebacterial species (including two with a reportedly markedly acidic cytoplasm). *A. aceti *has no C-A (or C-C) 'mismatches' in the 20 base pair positions of its tRNome, apart from a single universally conserved C1-A73 base pair found in all bacterial initiator tRNAs^Met ^(sequence data obtained from J. Kappock, pers. commun.; [79]). This may be because the internal pH of *A. aceti *can vary between pH 3.9-5.8 [33], causing fluctuations in protonation (and base pair formation) of any C-A (or C-C) mismatches present. Clearly this could cause problems for maintenance of the tRNA cloverleaf structure. A conclusion section has been added to the manuscript in response to this helpful suggestion.

Minor issues.

On p5 of the manuscript I think the reference numbering is out - reference 12 looks incorrect.

#### Authors' response

This reference has been corrected.

### Referee 3: Charles Carter, (nominated by David Ardell)

This paper put me in touch with a wide range of stimulating ideas, and hence I find it worthy of publication. This recommendation comes despite having been put off intensely by the arrogant statement that the RNA world hypothesis "... has wide, if not unanimous, support". I, for one, have never subscribed to this view of the origin of life, and I am by no means alone. The RNA world hypothesis is driven almost entirely by the flow of data from very high technology combinatorial libraries, whose relationship to the prebiotic world is anything but worthy of "unanimous support". There are several serious problems associated with it, and I view it as little more than a popular fantasy.

#### Authors' response

We thank the reviewer for drawing our attention to a misleading sentence on the RNA world hypothesis. We have always acknowledged the diversity of views concerning

the validity of this concept. Unintentional phrasing implied almost universal acceptance in the original submission and has now been changed. Indeed, having attended the recent ISSOL 2011 conference in Montpellier (HSB), we are certainly aware that not everyone is of one mind in this matter!

That said, this paper has the virtue of reviewing clearly a number of cogent arguments that do not depend on high contemporary technology. Many of these arguments - the pH-dependent stability of the ribose-phosphate backbone - are obvious and certainly not original. Yet, they form the core of the "hypothesis" offered by the authors. The paper is, nonetheless, an interesting review of a diverse collection of the experimental observations of others.

There are several points to be considered by the authors:

1. The notion that the prebiotic earth was warm is by no means strongly supported by astrophysical data from the past decade, and reference 5 should be provided with a more recent accompanying one.

#### Authors' response

As stated above in our response to the second reviewer, thermophile conditions are not required for our hypothesis of an acidic RNA world. Nevertheless, we have taken the reviewer's advice and added two more recent references: [6,7]. Although there has been a move away from the concept of a hot prebiotic Earth, Sleep *et al. *(2011) state, "*the origin of life under global thermophile conditions (e.g*. [1]) *remains a viable hypothesis*, as does the origin under clement conditions during latter stages of this process (italics added)" [6].

2. The discussion of base-mispairing in acidophiles at the end of the paper is potentially quite interesting. However, it is presented anecdotally, without statistical significance testing, which appears to be feasible in this case.

#### Authors' response

We thank the reviewer for this suggestion, and as a result the analysis now includes a one-sided Wilcoxon Rank Sum statistical significance test [65], which shows a significant correlation between an acidic internal pH and the occurrence of C-A mismatches. As an aside, it is interesting to note that the archaebacterial species analyzed have ~45 tRNAs in their tRNomes. With 20 base pairs per tRNA examined, this gives 900 base pair positions analyzed per species. Thus even in *F. acidarmanus*, which at 10 has the highest number of potentially protonatable C-A mismatches of the species examined, this represents only ~1% of these base pairs. This is interesting from an evolutionary point of view, as it suggests that the move into an acidic environment occurred relatively recently in evolutionary time (if *F. acidarmanus *was an extant member of an ancient lineage that inhabited a similarly acidic niche, one might have expected the proportion of protonatable base pairs in the tRNome to be considerably higher). It has been suggested that *F. acidarmanus*'s sister species *F. acidophilum *(which is similarly acidophilic and which has a high proportion (~86%) of metalloenzymes in its proteome [80]) is a relic from an ancestral acidic/high iron environment (unfortunately the *F. acidophilum *genome has not yet been sequenced). In light of the evidence presented here from *F. acidarmanus*, this would appear to be unlikely.

The relationship between an acidic internal pH and protonatable tRNA base pairs in eubacteria and eukaryotes may be slightly different to that found in archaebacteria: for example, *Escherichia coli *and *Saccharomyces cerevisiae *- both of which have a reported internal pH ~7 [81,82] - each have 5 C-A 'mismatches' in the same 20 base pairs of their tRNA genes (sequence data obtained from C. Marck, pers. commun.; [64]).

3. In as much as aminoacylation at low pH has "still to be verified" there is little scientific content to Figure 6.

#### Authors' response

Although aminoacylation at pH 4-5 has not been verified experimentally, Michael Yarus has made the following comment regarding the pH-dependence of the reaction rate of the original 5-nucleotide aminoacylating ribozyme reported in [48], a variant of which forms part of our proposed base-pair-specific aminoacylating ribozyme in Figure 6: "GUGGC is more or less log linear in pH, so 10x slower at pH 6 than 7" (M. Yarus, pers. commun.).

## References

[B1] Pace NR Origin of life--facing up to the physical setting Cell 1991 65 531 533 10.1016/0092-8674(91)90082-A 1709590

[B2] Abramov O Mojzsis SJ Microbial habitability of the Hadean Earth during the late heavy bombardment Nature 2009 459 419 422 10.1038/nature08015 19458721

[B3] Wilde SA Valley JW Peck WH Graham CM Evidence from detrital zircons for the existence of continental crust and oceans on the Earth 4.4 Gyr ago Nature 2001 409 175 178 10.1038/35051550 11196637

[B4] Russell MJ Hall AJ The emergence of life from iron monosulphide bubbles at a submarine hydrothermal redox and pH front J Geol Soc London 1997 154 377 402 10.1144/gsjgs.154.3.0377 11541234

[B5] Morse JW Mackenzie FT Hadean ocean carbonate geochemistry Aquat Geochem 1998 4 301 319 10.1023/A:1009632230875

[B6] Sleep NH Bird DK Pope EC Serpentinite and the dawn of life Philos Trans R Soc Lond B Biol Sci 2011 366 2857 2869 10.1098/rstb.2011.0129 21930576 PMC3158911

[B7] Sleep NH The Hadean-Archaean environment Cold Spring Harb Perspect Biol 2010 2 a002527 10.1101/cshperspect.a002527 20516134 PMC2869525

[B8] Martin W Russell MJ On the origin of biochemistry at an alkaline hydrothermal vent Philos Trans R Soc Lond B Biol Sci 2007 362 1887 1925 10.1098/rstb.2006.1881 17255002 PMC2442388

[B9] Martin W Baross J Kelley D Russell MJ Hydrothermal vents and the origin of life Nat Rev Microbiol 2008 6 805 814 18820700 10.1038/nrmicro1991

[B10] Järvinen P Oivanen M Lönnberg H Interconversion and phosphoester hydrolysis of 2',5'- and 3',5'-dinucleoside monophosphates: kinetics and mechanisms J Org Chem 1991 56 5396 5401 10.1021/jo00018a037

[B11] Jayasena VK Gold L *In vitro *selection of self-cleaving RNAs with a low pH optimum Proc Natl Acad Sci USA 1997 94 10612 10617 10.1073/pnas.94.20.10612 9380683 PMC23421

[B12] Burke DH Hoffman DC A novel acidophilic RNA motif that recognizes coenzyme A Biochemistry 1998 37 4653 4663 10.1021/bi972877p 9521786

[B13] Miyamoto Y Teramoto N Imanishi Y Ito Y *In vitro *evolution and characterization of a ligase ribozyme adapted to acidic conditions: effect of further rounds of evolution Biotechnol Bioeng 2005 90 36 45 10.1002/bit.20360 15723313

[B14] Miyamoto Y Teramoto N Imanishi Y Ito Y *In vitro *adaptation of a ligase ribozyme for activity under a low-pH condition Biotechnol Bioeng 2001 75 590 596 10.1002/bit.10033 11745135

[B15] Oivanen M Kuusela S Lönnberg H Kinetics and mechanisms for the cleavage and isomerization of the phosphodiester bonds of RNA by Brønsted acids and bases Chem Rev 1998 98 961 990 10.1021/cr960425x 11848921

[B16] Kühne H Joyce GF Continuous *in vitro *evolution of ribozymes that operate under conditions of extreme pH J Mol Evol 2003 57 292 298 10.1007/s00239-003-2480-z 14629039

[B17] Kumar RK Yarus M RNA-catalyzed amino acid activation Biochemistry 2001 40 6998 7004 10.1021/bi010710x 11401543

[B18] Chumachenko NV Novikov Y Yarus M Rapid and simple ribozymic aminoacylation using three conserved nucleotides J Am Chem Soc 2009 131 5257 5263 10.1021/ja809419f 19351205 PMC2750092

[B19] Di Giulio M On the origin of the transfer RNA molecule J Theor Biol 1992 159 199 214 10.1016/S0022-5193(05)80702-7 1294846

[B20] Bernhardt HS Tate WP Evidence from glycine transfer RNA of a frozen accident at the dawn of the genetic code Biol Direct 2008 3 53 10.1186/1745-6150-3-53 19091122 PMC2630981

[B21] Bernhardt HS Tate WP The transition from noncoded to coded protein synthesis: did coding mRNAs arise from stability-enhancing binding partners to tRNA? Biol Direct 2010 5 16 10.1186/1745-6150-5-16 20377916 PMC2859854

[B22] Wolfenden R The mechanism of hydrolysis of amino acyl RNA Biochemistry 1963 2 1090 1092 10.1021/bi00905a031 14087365

[B23] Schuber F Pinck M On the chemical reactivity of aminoacyl-tRNA ester bond. I. Influence of pH and nature of the acyl group on the rate of hydrolysis Biochimie 1974 56 383 390 10.1016/S0300-9084(74)80146-X 4853442

[B24] Varshney U Lee CP RajBhandary UL Direct analysis of aminoacylation levels of tRNAs *in vivo*. Application to studying recognition of *Escherichia coli *initiator tRNA mutants by glutaminyl-tRNA synthetase J Biol Chem 1991 266 24712 24718 1761566

[B25] Nissen P Kjeldgaard M Thirup S Polekhina G Reshetnikova L Clark BF Nyborg J Crystal structure of the ternary complex of Phe-tRNAPhe, EF-Tu, and a GTP analog Science 1995 270 1464 1472 10.1126/science.270.5241.1464 7491491

[B26] Pascal R Boiteau L Commeyras A From the prebiotic synthesis of α-amino acids towards a primitive translation apparatus for the synthesis of peptides Top Curr Chem 2005 259 69 122 10.1007/b136707

[B27] Ferris JP Usher DA Zubay G Origins of life Biochemistry 1983 Reading, MA: Addison-Wesley 1191 1241

[B28] Freeland SJ Knight RD Landweber LF Do proteins predate DNA? Science 1999 286 690 692 10.1126/science.286.5440.690 10577226

[B29] Varani G Exceptionally stable nucleic acid hairpins Annu Rev Biophys Biomol Struct 1995 24 379 404 10.1146/annurev.bb.24.060195.002115 7545040

[B30] Kua J Bada JL Primordial ocean chemistry and its compatibility with the RNA world Orig Life Evol Biosph 2011 41 553 558 22139511 10.1007/s11084-011-9250-5

[B31] Perez-Jimenez R Inglés-Prieto A Zhao ZM Sanchez-Romero I Alegre-Cebollada J Kosuri P Garcia-Manyes S Kappock TJ Tanokura M Holmgren A Sanchez-Ruiz JM Gaucher EA Fernandez JM Single-molecule paleoenzymology probes the chemistry of resurrected enzymes Nat Struct Mol Biol 2011 18 592 596 10.1038/nsmb.2020 21460845 PMC3087858

[B32] Xu SZ Sukumar P Zeng F Li J Jairaman A English A Naylor J Ciurtin C Majeed Y Milligan CJ Bahnasi YM Al-Shawaf E Porter KE Jiang LH Emery P Sivaprasadarao A Beech DJ TRPC channel activation by extracellular thioredoxin Nature 2008 451 69 72 10.1038/nature06414 18172497 PMC2645077

[B33] Menzel U Gottschalk G The internal pH of *Acetobacterium wieringae *and *Acetobacter aceti *during growth and production of acetic acid Arch Microbiol 1985 143 47 51 10.1007/BF00414767

[B34] Robertson MP Joyce GF Atkins JF, Gesteland RF, Cech TR The origins of the RNA world RNA Worlds 2010 Cold Spring Harbor, NY: Cold Spring Harbour Press 21 42

[B35] Inoue T Joyce GF Grzeskowiak K Orgel LE Brown JM Reese CB Template-directed synthesis on the pentanucleotide CpCpGpCpC J Mol Biol 1984 178 669 676 10.1016/0022-2836(84)90244-4 6092644

[B36] Acevedo OL Orgel LE Non-enzymatic transcription of an oligodeoxynucleotide 14 residues long J Mol Biol 1987 197 187 193 10.1016/0022-2836(87)90117-3 3681994

[B37] Romby P Westhof E Moras D Giegé R Houssier C Grosjean H Studies on anticodon-anticodon interactions: hemi-protonation of cytosines induces self-pairing through the GCC anticodon of *E. coli *tRNA-Gly J Biomol Struct Dyn 1986 4 193 203 2856023 10.1080/07391102.1986.10506339

[B38] Mariappan SV Catasti P Chen X Ratliff R Mazes RK Bradbury EM Gupta G Solution structures of the individual single strands of the fragile × DNA triplets (GCC)*_n_*.(GGC)*_n_* Nucleic Acids Res 1996 24 784 792 10.1093/nar/24.4.784 8604324 PMC145702

[B39] Durant PC Davis DR Stabilization of the anticodon stem-loop of tRNALys,3 by an A+-C base-pair and by pseudouridine J Mol Biol 1999 285 115 131 10.1006/jmbi.1998.2297 9878393

[B40] Meroueh M Chow CS Thermodynamics of RNA hairpins containing single internal mismatches Nucleic Acids Res 1999 27 1118 1125 10.1093/nar/27.4.1118 9927746 PMC148293

[B41] Chirkova A Erlacher MD Clementi N Zywicki M Aigner M Polacek N The role of the universally conserved A2450-C2063 base pair in the ribosomal peptidyl transferase center Nucleic Acids Res 2010 38 4844 4855 10.1093/nar/gkq213 20375101 PMC2919715

[B42] Venditti V Clos L Niccolai N Butcher SE Minimum-energy path for a U6 RNA conformational change involving protonation, base-pair rearrangement and base flipping J Mol Biol 2009 391 894 905 10.1016/j.jmb.2009.07.003 19591840 PMC2799254

[B43] Bink HH Hellendoorn K van der Meulen J Pleij CW Protonation of non-Watson-Crick base pairs and encapsidation of turnip yellow mosaic virus RNA Proc Natl Acad Sci USA 2002 99 13465 13470 10.1073/pnas.202287499 12361978 PMC129696

[B44] Schimmel P Giegé R Moras D Yokoyama S An operational RNA code for amino acids and possible relationship to genetic code Proc Natl Acad Sci USA 1993 90 8763 8768 10.1073/pnas.90.19.8763 7692438 PMC47440

[B45] Holland JA Hoffman DW Structural features and stability of an RNA triple helix in solution Nucleic Acids Res 1996 24 2841 2848 10.1093/nar/24.14.2841 8759020 PMC146013

[B46] Shefer K Brown Y Gorkovoy V Nussbaum T Ulyanov NB Tzfati Y A triple helix within a pseudoknot is a conserved and essential element of telomerase RNA Mol Cell Biol 2007 27 2130 2143 10.1128/MCB.01826-06 17210648 PMC1820488

[B47] Mergny JL Sun JS Rougée M Montenay-Garestier T Barcelo F Chomilier J Hélène C Sequence specificity in triple-helix formation: experimental and theoretical studies of the effect of mismatches on triplex stability Biochemistry 1991 30 9791 9798 10.1021/bi00104a031 1911764

[B48] Turk RM Chumachenko NV Yarus M Multiple translational products from a five-nucleotide ribozyme Proc Natl Acad Sci USA 2010 107 4585 4589 10.1073/pnas.0912895107 20176971 PMC2826339

[B49] Jühling F Mörl M Hartmann RK Sprinzl M Stadler PF Pütz J tRNAdb 2009: compilation of tRNA sequences and tRNA genes Nucleic Acids Res 2009 37 D159 162 10.1093/nar/gkn772 18957446 PMC2686557

[B50] Clark BC Primeval procreative comet pond Orig Life Evol Biosph 1988 18 209 238 10.1007/BF01804671 3226718

[B51] Chen IA Szostak JW Membrane growth can generate a transmembrane pH gradient in fatty acid vesicles Proc Natl Acad Sci USA 2004 101 7965 7970 10.1073/pnas.0308045101 15148394 PMC419540

[B52] Namani T Deamer DW Stability of model membranes in extreme environments Orig Life Evol Biosph 2008 38 329 341 10.1007/s11084-008-9131-8 18560991

[B53] de Duve C Blueprint for a Cell: The Nature and Origin of Life 1991 Burlington, North Carolina: Neil Patterson Publishers, Carolina Biological Supply Company 179

[B54] Engelhart AE Hud NV Primitive genetic polymers Cold Spring Harb Perspect Biol 2010 2 a002196 10.1101/cshperspect.a002196 20462999 PMC2982173

[B55] Domingo E Escarmís C Sevilla N Moya A Elena SF Quer J Novella IS Holland JJ Basic concepts in RNA virus evolution FASEB J 1996 10 859 864 8666162 10.1096/fasebj.10.8.8666162

[B56] Ruepp A Graml W Santos-Martinez ML Koretke KK Volker C Mewes HW Frishman D Stocker S Lupas AN Baumeister W The genome sequence of the thermoacidophilic scavenger *Thermoplasma acidophilum* Nature 2000 407 508 513 GenBank: AL139299.1 10.1038/35035069 11029001

[B57] Michels M Bakker EP Generation of a large, protonophore-sensitive proton motive force and pH difference in the acidophilic bacteria *Thermoplasma acidophilum *and *Bacillus acidocaldarius* J Bacteriol 1985 161 231 237 2981803 10.1128/jb.161.1.231-237.1985 PMC214861

[B58] Schneider KL Pollard KS Baertsch R Pohl A Lowe TM The UCSC Archaeal Genome Browser Nucleic Acids Res 2006 34 Database D407 D410 *F. acidarmanus *June 2005 draft (ferrAcid1) assembly; GenBank: AABC05000000 16381898 10.1093/nar/gkj134 PMC1347496

[B59] Chan PP Lowe TM GtRNAdb: a database of transfer RNA genes detected in genomic sequence Nucleic Acids Res 2009 37 Database D93 D97 18984615 10.1093/nar/gkn787 PMC2686519

[B60] Macalady JL Vestling MM Baumler D Boekelheide N Kaspar CW Banfield JF Tetraether-linked membrane monolayers in *Ferroplasma *spp: a key to survival in acid Extremophiles 2004 8 411 419 10.1007/s00792-004-0404-5 15258835

[B61] van de Vossenberg Driessen AJ Zillig W Konings WN Bioenergetics and cytoplasmic membrane stability of the extremely acidophilic, thermophilic archaeon *Picrophilus oshimae* Extremophiles 1998 2 67 74 10.1007/s007920050044 9672680

[B62] She Q Singh RK Confalonieri F Zivanovic Y Allard G Awayez MJ Chan-Weiher CC Clausen IG Curtis BA De Moors A Erauso G Fletcher C Gordon PM Heikamp-de Jong I Jeffries AC Kozera CJ Medina N Peng X Thi-Ngoc HP Redder P Schenk ME Theriault C Tolstrup N Charlebois RL Doolittle WF Duguet M Gaasterland T Garrett RA Ragan MA Sensen CW Van der Oost J The complete genome of the crenarchaeon *Sulfolobus solfataricus *P2 Proc Natl Acad Sci USA 2001 98 7835 7840 GenBank: AE006641.1 10.1073/pnas.141222098 11427726 PMC35428

[B63] Moll R Schäfer G Chemiosmotic H^+ ^cycling across the plasma membrane of the thermoacidophilic archaebacterium *Sulfolobus acidocaldarius* FEBS Letters 1988 232 359 363 10.1016/0014-5793(88)80769-5

[B64] Marck C Grosjean H tRNomics: analysis of tRNA genes from 50 genomes of Eukarya, Archaea, and Bacteria reveals anticodon-sparing strategies and domain-specific features RNA 2002 8 1189 1232 10.1017/S1355838202022021 12403461 PMC1370332

[B65] Mann HB Whitney DR On a test of whether one of two random variables is stochastically larger than the other Ann Math Statist 1947 18 50 60 10.1214/aoms/1177730491

[B66] Golyshina OV Golyshin PN Timmis KN Ferrer M The 'pH optimum anomaly' of intracellular enzymes of *Ferroplasma acidiphilum* Environ Microbiol 2006 8 416 425 10.1111/j.1462-2920.2005.00907.x 16478448

[B67] Rajamani S Vlassov A Benner S Coombs A Olasagasti F Deamer D Lipid-assisted synthesis of RNA-like polymers from mononucleotides Orig Life Evol Biosph 2008 38 57 74 10.1007/s11084-007-9113-2 18008180

[B68] Olasagasti F Kim HJ Pourmand N Deamer DW Non-enzymatic transfer of sequence information under plausible prebiotic conditions Biochimie 2011 93 556 561 10.1016/j.biochi.2010.11.012 21130835

[B69] Brasier MD Matthewman R McMahon S Wacey D Pumice as a remarkable substrate for the origin of life Astrobiology 2011 11 725 735 10.1089/ast.2010.0546 21879814

[B70] Lathe R Fast tidal cycling and the origin of life Icarus 2003 168 18 22

[B71] Lathe R Tidal chain reaction and the origin of replicating biopolymers Int J Astrobiol 2005 4 19 31 10.1017/S1473550405002314

[B72] Nilson FP Possible impact of a primordial oil slick on atmospheric and chemical evolution Orig Life Evol Biosph 2002 32 247 253 10.1023/A:1016577923630 12227429

[B73] Guo F Cech TR Evolution of *Tetrahymena *ribozyme mutants with increased structural stability Nat Struct Biol 2002 9 855 861 12368901 10.1038/nsb850

[B74] Rhee S Han Z Liu K Miles HT Davies DR Structure of a triple helical DNA with a triplex-duplex junction Biochemistry 1999 38 16810 16815 10.1021/bi991811m 10606513

[B75] Strickler SS Gribenko AV Gribenko AV Keiffer TR Tomlinson J Reihle T Loladze VV Makhatadze GI Protein stability and surface electrostatics: a charged relationship Biochemistry 2006 45 2761 2766 10.1021/bi0600143 16503630

[B76] Galtier N Tourasse N Gouy M A nonhyperthermophilic common ancestor to extant life forms Science 1999 283 220 221 10.1126/science.283.5399.220 9880254

[B77] Boussau B Blanquart S Necsulea A Lartillot N Gouy M Parallel adaptations to high temperatures in the Archaean eon Nature 2008 456 942 945 10.1038/nature07393 19037246

[B78] Arrhenius G Bada JL Joyce GF Lazcano A Miller S Orgel LE Origin and ancestor: separate environments Science 1999 283 792 10049121 10.1126/science.283.5403.791c

[B79] Francois JA Kappock TJ Alanine racemase from the acidophile *Acetobacter aceti* Protein Expr Purif 2007 51 39 48 [http://www.ncbi.nlm.nih.gov/pubmed/16843006,http://dx.doi.org/10.1016/j.pep.2006.05.016] 10.1016/j.pep.2006.05.016 16843006

[B80] Ferrer M Golyshina OV Beloqui A Golyshin PN Timmis KN The cellular machinery of Ferroplasma acidiphilum is iron-protein-dominated Nature 2007 445 91 94 10.1038/nature05362 17203061

[B81] Hickey EW Hirshfield IN Low-pH-induced effects on patterns of protein synthesis and on internal pH in *Escherichia coli *and *Salmonella typhimurium* Appl Environ Microbiol 1990 56 1038 1045 2187401 10.1128/aem.56.4.1038-1045.1990 PMC184340

[B82] Dombek KM Ingram LO Ethanol production during batch fermentation with *Saccharomyces cerevisiae*: changes in glycolytic enzymes and internal pH Appl Environ Microbiol 1987 53 1286 1291 3300550 10.1128/aem.53.6.1286-1291.1987 PMC203856

